# Maternal Obesity and Energy Intake as Risk Factors of Pregnancy-induced Hypertension among Iranian Women

**Published:** 2014-09

**Authors:** Elham Kazemian, Gity Sotoudeh, Ahmad Reza Dorosty-Motlagh, Mohammad Reza Eshraghian, Minoo Bagheri

**Affiliations:** ^1^Department of Community Nutrition, School of Nutritional Sciences and Dietetics, Tehran, Iran; ^2^Department of Statistics and Epidemiology, School of Public Health, Tehran University of Medical Sciences, Tehran, Iran

**Keywords:** Body mass index, Energy intake, Gestational hypertension, Gestational weight gain, Pregnancy, Pre-pregnancy, Iran

## Abstract

Pregnancy-induced hypertension is causing striking maternal, foetal and neonatal mortality and morbidity in the world. A case-control study was conducted on 113 women with gestational hypertension and 150 healthy pregnant women at Shahid Akbarabadi Hospital of obstetrics and gynaecology in south of Tehran. Women who were obese (OR 4.44; 95% CI 1.84-10.72) before pregnancy were more likely to develop gestational hypertension. Proportion of having excessive gestational weight gain was positively and significantly associated with development of gestational hypertension (OR 2.70; 95% CI 1.19-6.13). Furthermore, findings revealed that women who were in the highest quartile of mid-arm-circumference had a 3-fold increased risk of gestational hypertension compared to women in the lowest quartile (OR 8.93; 95% CI 2.16-36.93). We found that having been in the highest quartile of energy intake positively correlated with increased risk of gestational hypertension (OR 9.66; 95% CI 3.30-28.21). The results suggest pre-pregnancy obesity, excessive gestational weight gain, and increased intake of energy as potential risk factors of developing gestational hypertension.

## INTRODUCTION

Pregnancy-induced hypertension (PIH) is an abnormality causing striking maternal, foetal and neonatal mortality and morbidity both in developed and developing countries (1). PIH is observed in forms of gestational hypertension, pre-eclampsia, and eclampsia (1). Pre-eclampsia and gestational hypertension are found in 5-10% of pregnancies in the world (2). Increase in caesarean section, abruption of premature placenta, preterm delivery, low birthweight, stillbirth, acute renal failure, and intravascular coagulation were more frequently observed in women who developed hypertensive disorders of pregnancy (3-4). Recent studies have indicated higher risk of PIH among women with family history of hypertension, previous history of pregnancy-induced hypertension, pre-exciting diabetes, gestational diabetes mellitus, maternal age ≥40 years, multiple pregnancies, nulliparity, and pre-pregnancy obesity (5-10). Some prior studies have suggested that higher pre-pregnancy body mass index is associated with increased risk of gestational hypertension and pre-eclampsia (11-15). However, there are a few studies in which this association was not observed (16). Also, excessive gestational weight gain has been proposed as a risk factor of hypertensive disorders of pregnancy in some studies (17-20). PIH is accompanied with endothelial dysfunction, oxidative stress, and inflammatory responses (1). It has been claimed that plasma C-reactive protein concentration, which may be involved in an aetiology of hypertensive disorder of pregnancy increased in obesity. Furthermore, some evidences have indicated that obesity increased endothelial function and prompted systematic inflammatory responses associated with atherosclerosis, which could play a role in PIH (21). However, previous studies are limited by improper classification of gestational weight gain sometimes by restricting study population to one BMI category and, also, none of these studies evaluated energy intake of subjects, alongside other measurements, which defiantly led to more accurate determination (19-20). Although risk factors of developing gestational hypertension may differ among various ethnic groups (22), there are a few data with regard to this issue in Iranian population. So, the aim of the present observational study was to compare pre-pregnancy body mass index, mid-arm-circumference, gestational weight gain, and energy intake of women who developed gestational hypertension with those of healthy pregnant women.

## MATERIALS AND METHODS

### Subjects and study design

The current research was a case-control study which has been carried out in Shahid Akbarabadi Hospital of obstetrics and gynaecology in south of Tehran (This is a referral hospital; many pregnant women had been referred to this centre) from January through May 2011. Patients referred to the hospital, diagnosed with gestational hypertension by physicians, were assessed to determine whether they had met exclusion criteria of the present study or not. Having multiple gestations, chronic hypertension, diabetes, cardiovascular or renal diseases were considered exclusion criteria in the present investigation. Subjects who had these problems were not included in the study. Also, pregnant women whose first antenatal care (ANC) visits were made after 12 weeks of gestation were excluded. Systolic blood pressure ≥140 mmHg or diastolic blood pressure ≥90 mmHg, which occurred after 20 weeks of gestation for the first time was defined as gestational hypertension according to the National High Blood Pressure Education Program Working Group (23). Controls were women without gestational hypertension, who were referred to the clinic of this hospital for their antenatal care visits or were hospitalized at prenatal section of this centre for other reasons and were matched with cases for gestational age. The same procedure and exclusion criteria were applied for recruitment of cases. Sample-size was calculated on the basis of one previous study that assessed nutritional status of pre-eclamptic women in Iranian population. We assumed that if the pre-pregnancy BMIs of pre-eclamptic women were different from women without pre-eclampsia, the hypothesis suggesting equal pre-pregnancy BMIs between cases and controls could be rejected with power of 80% and the significance level of 1.96. The following formula was applied to calculate sample-size in the present study:


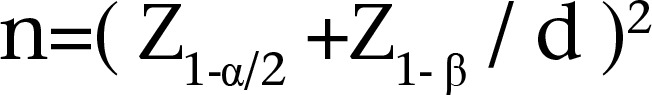


where n=sample-size; Z_1-α/2_=1.96; Z1_1-β_= 0.84; α = 0.05; β = 80%; d=X1-X2/2 δ^2^

X1-X2 being difference in means of the pre-pregnancy BMIs of pre-eclamptic women from women without pre-eclampsia and δ being standard deviation of independent sample.

Ultimately, this study has been conducted on 113 women with gestational hypertension and 150 healthy pregnant women. The study was approved by the Ethics Committee of Tehran University of Medical Sciences, and all participants were informed, and then they provided written consent. Subjects were interviewed by trained interviewers for sociodemographic information, including maternal age, gestational age, parity, abortion, and gravidity, pregnancy interval, family history of hypertension, previous pregnancy-induced hypertension, sleeping hours per day, number of ANC visits, education, and occupation.

### Anthropometric measurements

Pre-pregnancy weights were self-reported, and patients were asked to report their weights at the last menstrual period at the time of data collection. Weight at the first ANC visit was registered from medical records and compared with pre-pregnancy weight. If subjects did not meet the criteria of 0.2-3.8 kg weight gain during the first four-week period of pregnancy as reported by previous research (28), they were excluded from the study. Heights were measured by Seca stadiometer in a position while the persons were standing directly, with feet together and without shoes. Heels, buttocks, and upper back were in contact with the wall when the measurement was taken. Pre-pregnancy BMI [weight (kg)/height (m)^2^] was calculated based upon measured height and self-reported pre-pregnancy weight. BMI was categorized according to 2009 IOM classification: underweight (BMI <18.5), normal weight (BMI 18.5-24.9), overweight (BMI 25.0-29.9), and obese (BMI ≥30.0) (24). In addition, weights were measured at the time of data collection by portable digital Seca scale in a condition where subjects were without shoes and minimally clothed. Gestational weight gain was calculated by subtracting pre-pregnancy weight from the weight which was measured at the time of data collection. Also, gestational weight gain proportion was derived by observed gestational weight gain divided by expected weight gain at their gestational age. According to 2009 IOM guidelines, weight gains of 0.44 to 0.58 kg/week for women with a pre-pregnancy BMI of less than 18.5 kg/m^2^ 0.35 to 0.50 kg for women with a pre-pregnancy BMI of 18.5 to 24.9 kg/m^2^ and 0.23 to 0.33 kg for women with a pre-pregnancy BMI of 25.0 to 29.9 kg/m^2^ are suggested. The recommendation for obese women (BMI >29.9 kg/m^2^) is 0.17 to 0.27 kg/week. It should be noted that recommended weight gains at the first trimester for women with a pre-pregnancy BMI of less than 30 kg/m^2^ and women with a pre-pregnancy BMI of more than 30 kg/m^2^ are 2 kg and 1.5 kg respectively (24). Mid-arm-circumference is the circumference of the left upper arm, measured at midpoint of the distance from the acromion process of the shoulder to the tip of the olecranon process of the mid-elbow.

### Assessment of energy intake

A semi-quantitative food frequency questionnaire (SFFQ) was employed to assess energy intakes of subjects. The average frequency of consumption of food “during three months before”, coinciding with their first mid-pregnancy, was recorded. The SFFQ used consisted of 148 items of food, with standard serving-size validated in the Tehran lipid and glucose study (25). Finally, energy intakes of participants were calculated by Nutritionist III software modified for Iranian foods.

### Statistical analysis

Mean levels of quantitative variables were estimated for women developing gestational hypertension and healthy pregnant women. Normal distribution of each variable was assessed by Kolmogrove-Smirnov test. Quantitative variables between groups were compared by Student's *t*-test or Mann-Whitney U-test whereas chi-square test was employed to compare the qualitative variables. Multivariable logistic regression was used in determining an association of pre-pregnancy BMI gestational weight gain, mid-arm-circumference and energy intake with development of gestational hypertension. Any covariate that showed significant difference between two groups was retained in the final model. Indeed, estimates were matched for age, abortion, gravidity, pregnancy interval, family history of hypertension, hypertension in previous pregnancy, and education. Odds ratios (ORs) and 95% confidence intervals (CIs) as well as the p values were reported. Age, abortion, gravidity, pregnancy interval, pre-pregnancy BMI, family history of hypertension, hypertension in previous pregnancy, and education were included as covariates in the final model. Analysis of all data was performed by using SPSS (version 11.5) (SPSS Inc., Chicago IL, USA).

## RESULTS

Sociodemographic features of participants are shown in Table 1. The mean age, parity, abortion, gravidity, and pregnancy interval of healthy pregnant women were significantly lower than women with gestational hypertension (p value <0.05). Women developing gestational hypertension were more prone to having family history of hypertension and previous history of gestational hypertension (p value <0.001). We found that 34% of women developing gestational hypertension and 23% of healthy pregnant women were non-literate, or had primary education (p value <0.05). Number of ANC visits, sleeping hours, occupation, and nulliparity were not significantly associated with risk of gestational hypertension.

Table 2 shows anthropometric measurements and energy intake of subjects. All anthropometric measurements, excluding height, in women who developed gestational hypertension, were significantly higher than healthy pregnant women (p value <0.05). Also, higher intake of energy was observed in case group compared to the controls (p value <0.05).

Adjusted odds ratio in the different pre-pregnancy BMI groups as well as different gestational weight gain proportion groups, mid-arm-circumference and energy intake quartile are shown in Table 3. Women of normal weight were considered the reference group. Women who were obese (OR 4.44; 95% CI 1.84-10.72) before becoming pregnant were more likely to develop gestational hypertension compared to those who had normal pre-pregnancy BMI. Additionally, having excessive gestational weight gain was positively and significantly associated with development of gestational hypertension (OR 2.70; 95% CI 1.19-6.13). Furthermore, findings of present study revealed that women who were in the highest quartile of mid-arm-circumference had an almost 9-fold increased risk of gestational hypertension compared to women in the lowest quartile (OR 8.93; 95% CI 2.16-36.93). Regarding energy intake, the study revealed that women of the highest quartile of energy intake were approximately 9 times more likely to develop gestational hypertension opposed to women in the lowest quartile (OR 9.66; 95% CI 3.30-28.21).

## DISCUSSION

In this case-control study, we found that patients with pre-pregnancy BMI more than 30 kg/m^2^ had a nearly 4.5-fold risk of developing gestational hypertension compared to pregnant women whose pre-pregnancy BMIs were in the normal range. Furthermore, subjects with gestational weight gain of more than recommended value had an approximate 3-fold risk of gestational hypertension compared to those who had normal gestational weight gain. Also, the result of present study revealed a somewhat higher risk of gestational hypertension with increased mid-arm-circumference and energy intake during pregnancy. Totally, our findings have suggested obesity as a risk factor of developing gestational hypertension.

**Table 1.** Sociodemographic characteristics of participants who developed gestational hypertension and of healthy pregnant women

Characteristics	Pregnant women with gestational hypertension (N=113)	Healthy pregnant women (N=150)	p value^a^
	Mean±SD		
Age (years)	28.73±6.04	25.36±4.84	<0.001
Parity (N)	2.75±0.91	2.53±0.82	0.033
Gravidity (N)	0.74±0.91	0.51±0.74	0.038
Pregnancy interval (years)	3.89±5.15	2.31±3.52	0.029
Abortion	0.46±0.69	0.17±0.50	<0.001
Number of ANC visits	9.15±4.39	9.03±8.50	0.136
Sleeping hours per day	8.73±2.86	8.81±2.64	0.678
Gestational age (weeks)	33.39±4.67	33.22±3.73	0.321
	N (percentage)		
Family history of hypertension			
No	68 (60.2)	130 (86.7)	<0.001
Yes	35 (31.0)	10 (6.7)	
Don't know	10 (8.8)	10 (6.7)	
Hypertension in previous pregnancy			
No	45 (39.8)	68 (45.3)	<0.001
Yes	16 (14.2)	1 (0.7)	
Don't know or first pregnancy	52 (46.0)	81 (54.0)	
Education			
Uneducated or primary school	34 (30.1)	23 (15.3)	
Junior high school	25 (22.1)	45 (30.0)	
Diploma	46 (40.7)	66 (44.0)	0.029
College	8 (7.1)	16 (10.7)	
Occupation			
Employed	9 (8.0)	9 (6.0)	
Unemployed	104 (92.0)	141 (94.0)	0.624
Nulliparity	58 (51.3)	91 (60.7)	0.286

ap value for quantitative variables resulted from Mann-Whitney U-test, and p values for qualitative variables resulted from chi-square test

**Table 2.** Mean levels of anthropometric measurements and energy intake in pregnant women who developed gestational hypertension and healthy pregnant women

Anthropometric measurement	Pregnant women with gestational hypertension (N=113)	Healthy pregnant women (N=150)	p value^a^
	Mean±SD	Mean±SD	
Pre-pregnancy weight (kg)	72.35±16.24	59.84±12.08	<0.001
Weight in first ANC visit (kg)	74.37±15.51	61.67±12.59	<0.001
Height (cm)	157.97±5.90	158.92±6.12	<0.001
Pre-pregnancy BMI (kg/m^2^)	28.97±6.31	23.70±4.64	<0.001^b^
Gestational weight gain (kg)	14.08±8.28	11.69±5.16	0.023
Gestational weight gain proportion	1.76±1.05	1.24±0.63	<0.001
Mid-arm-ircumference (cm)	33.51±9.97	27.80±3.61	<0.001 <0.001^b^
Energy intake (kcal)	2,794.1±537.8	2,430.8±556.4

^a^p value resulted from Mann-Whitney U-test;

^b^p value resulted from Student's *t*-test

**Table 3.** Adjusted odds ratio (AOR)^a^ for the effect of pre-pregnancy BMI, gestational weight gain proportion, mid-arm-circumference and energy intake on development of gestational hypertension

Variable	Gestational hypertension vs normal blood pressure		
	Odds ratio	95% confidence interval	p value
Pre-pregnancy BMI (kg/m^2^)			
Normal weight (18.5-24.9 kg/m^2^)	1	1	
Underweight (<18.5 kg/m^2^)	0.10	0.01-0.94	0.044
Overweight (25.0-29.9 kg/m^2^)	1.69	0.79-3.60	0.171
Obese (≥30.0 kg/m^2^)	4.44	1.84-10.72	0.001
Gestational weight gain proportion			
Adequate	1	1	
Inadequate	0.38	0.10-1.42	0.152
Excessive	2.70	1.19-6.13	0.017
Mid-arm-circumference (cm)			
<26	1	1	
26.1-29	1.86	0.60-5.76	0.280
29.1-32	4.30	1.18-15.67	0.027
>32	8.93	2.16-36.93	0.002
Energy intake (kcal)			
<2,154	1	1	
2,154-2,561	0.652	0.26-1.73	0.575
2,562-3,036	1.14	1.04-1.25	0.005
>3,036	9.66	3.30-28.21	<0.001

^a^Estimates are adjusted for age, abortion, gravidity, pregnancy interval, family history of hypertension, hypertension in previous pregnancy, and education. Adjusted odds ratio for pre-pregnancy BMI, gestational weight gain, and mid-arm-circumference resulted from separate models

The results of the present research associated with pre-pregnancy BMI are in the same direction with the observed relationship between pre-pregnancy BMI and PIH in other studies conducted in different countries (12-13,15,17,19-21,26-27). However, in one study conducted by Tabandeh *et al*. in Iranian population, no significant association was found between pre-pregnancy BMI and the risk of pre-eclampsia (16). Inadequate number of patients developing pre-eclampsia was one of the main limitations of this study. In a great number of studies, BMI was classified according to those issued in 1990 IOM guidelines, which differ in BMI categories with the new guidelines of this institution. Also, newly-published guidelines of IOM recommend relatively narrow range of gestational weight gain for obese women. In the present investigation, BMI classification and judgement on gestational weight gain were made in accordance with the recently-published guidelines of IOM and, afterwards, the risk of developing gestational hypertension was assessed for each group.

Few studies have investigated the association of gestational weight gain and hypertensive disorders of pregnancy. However, some previous studies have indicated the direct association between gestational weight gain and gestational hypertension and pre-eclampsia (16-20). Chen *et al*. reported that women with a gestational weight gain of 0.50 kg per week or greater were at increased risk of gestational hypertension (19). Moreover, Fortner *et al*., in a study which was conducted on women from Latin America, observed that excessive gestational weight gain increased the risk of gestational hypertension and pre-eclampsia nearly 4 and 3 folds respectively (20).

We calculated gestational weight gain by using measured weight and self-reported pre-pregnancy weight. An overall correlation coefficient of 0.99 between self-reported and measured pre-pregnancy weight was noted by Oken *et al.* (28). However, a lot of inter-individual variations account for the validity of self-reported pre-gravid weight. Furthermore, we found mean maternal weight gain of 1.9 kg (data not shown) during the early stages of pregnancy, calculated by self-reported pre-gravid weight and measured weight at the first ANC visit which was between 8 and 12 weeks of gestation. Pregnant women were reported to have gained anything from 0.2 kg to 3.8 kg during the first four-week period of pregnancy in studies that measured pre-pregnancy weights (29). Thus, mean maternal weight gain of 1.9 kg (data not shown) in early pregnancy in the present investigation was within the range of mean weight gain in early pregnancy reported by previous studies (29). Indeed, mixture of methods was utilized to minimize this bias.

It could not be decided whether oedema contributed in the observed increase in gestational weight and mid-arm-circumference in patients developing gestational hypertension or not. Since that, oedema has also been observed in up to 80% of normal pregnancies; oedema as a criterion for diagnosing hypertensive disorder of pregnancy was eliminated (23,30-33). However, we did neither weigh the subjects prior to the outset of gestational hypertension nor information with regard to presence of oedema in cases and controls was available. In view of the fact that this study had a case-control design and cause-and-effect relationship is scarcely determined in case-control studies, it is difficult to interpret whether observed increase in gestational weight and mid-arm-circumference among hypertensive women resulted from fluid retention or increase of fat or muscle.

A highly important factor assessed in the present study, which has not been investigated in prior study inspecting obesity as a potential risk factor of developing gestational hypertension was energy intake of participants, which assist us to draw a conclusion. We found that not only did higher gestational weight gain proportion increased the risk of pregnancy-induced hypertension but also women who were in the highest quartile of energy intake had increased risk of developing this syndrome. This result directed us to conclude that observed higher gestational weight gain proportion among cases were originated from higher intake of energy during pregnancy, supporting that increase of maternal fat or muscle contributed in the aetiology of gestational hypertension.

Mahomed *et al*. reported that women in the highest quintile of mid-arm-circumference (28-39 cm) were more likely (4.4 times) to develop pre-eclampsia compared to women in the lowest quintile (21-23 cm), which is consistent with the result of the present study (27).

The possible mechanisms by which obesity could induce hypertensive disorders of pregnancy are not well-understood. Nevertheless, some predictable mechanisms through which hypertension were prompted might be the unfavourable effects of changes, such as insulin resistance and elevation of cholesterol and leptin levels, which have been observed in obese persons with blood pressure (34-35). In addition, both obesity and hypertensive disorders of pregnancy, accompanied with oxidative stress, elevated inflammatory markers, and dislipidaemia (36).

### Strengths and limitations

One limitation of the present study is its case-control design, in which cause-and-effect relationship is not distinguished. Also, we have not measured pre-gravid weights objectively, and we relied on self-reported pre-pregnancy weight. An important strength of the present investigation was to assess energy intake of subjects as well as anthropometric measurements, which helped us interpret results of the study more precisely. In addition, in this study, both pre-pregnancy BMI and weight gain during pregnancy were assessed that were conducted in a few previous studies. Additionally, the new guidelines of IOM were used in classifying pre-pregnancy BMI and interpret gestational weight gain.

### Conclusions

Pre-pregnancy obesity, excessive gestational weight gain, and higher energy intake during pregnancy were noted as modifiable risk factors of developing gestational hypertension in the current investigation. It can be suggested that experimental research should be designed to examine whether improvement of these factors can reduce the risk of gestational hypertension.

## ACKNOWLEDGEMENTS

The present study was supported by the Tehran University of Medical Sciences, Iran, Tehran. We gratefully acknowledge the contributions made to the research by Shahid Akbarabadi Hospital staff. We are also thankful to the women who participated in the survey.
